# A Splice Site Variant in *ADAMTS3* Is the Likely Causal Variant for Pulmonary Hypoplasia with Anasarca in Persian/Persian-Cross Sheep

**DOI:** 10.3390/ani14192811

**Published:** 2024-09-29

**Authors:** Shernae A. Woolley, Bethany Hopkins, Mehar S. Khatkar, Ian V. Jerrett, Cali E. Willet, Brendon A. O’Rourke, Imke Tammen

**Affiliations:** 1 Sydney School of Veterinary Science, Faculty of Science, The University of Sydney, Sydney, NSW 2006, Australia; 2 Agriculture Victoria, AgriBio Centre, Bundoora, VIC 3083, Australia; 3 Sydney Informatics Hub, Core Research Facilities, The University of Sydney, Sydney, NSW 2006, Australia; 4 Elizabeth Macarthur Agricultural Institute, NSW Department of Primary Industries and Regional Development, Menangle, NSW 2568, Australia; brendon.orourke@dpi.nsw.gov.au

**Keywords:** hydrops fetalis, sheep, recessive, inherited disease, whole genome sequencing

## Abstract

**Simple Summary:**

Pulmonary hypoplasia with anasarca, or hydrops fetalis, is a fatal inherited disease reported in several species. Affected fetuses are stillborn and present with the buildup of fluid within body cavities and tissues and under-developed lungs and lymph tissues. The enlarged size of the fetus frequently results in difficult or obstructed labor. This is the first report of the disease in three flocks of Persian/Persian-cross sheep in Australia. We describe the pathology of affected fetuses and conducted genetic research that identified and validated a variant in the *ADAMTS3* gene as the likely cause for this recessive disease in these sheep. A diagnostic DNA test was developed which allows selective breeding to reduce the risk of affected animals being born.

**Abstract:**

Pulmonary hypoplasia with anasarca, or hydrops fetalis, is characterized by stillbirth, diffuse oedema, and generalized lymph node hypoplasia. The enlarged fetus frequently causes dystocia. The disease has been reported in cattle and sheep as an inherited condition with a recessive mode of inheritance. This is the first report of the disease in Persian/Persian-cross sheep in Australia. Affected fetuses were reported from three flocks, and a total of eleven affected, eleven obligate carrier, and 188 related Persian/Persian-cross animals were available for analysis, as well as unrelated control animals. SNP genotyping revealed a region of homozygosity in affected animals on ovine chromosome six, which contained the functional candidate gene *ADAMTS3*. Whole genome sequencing of two affected fetuses and one obligate carrier ewe revealed a single nucleotide deletion, ENSOARG00000013204:g.87124344delC, located 3 bp downstream from a donor splice site region in the *ADAMTS3* gene. Sanger sequencing of cDNA containing this variant further revealed that it is likely to introduce an early splice site in exon 14, resulting in a loss of 6 amino acids at the junction of exon 14 and intron 14/15. A genotyping assay was developed, and the ENSOARG00000013204:g.87124344delC segregated with disease in 209 animals, allowing for effective identification of carrier animals.

## 1. Introduction

Pulmonary hypoplasia with anasarca (PHA) or hydrops fetalis is an inherited condition affecting fetuses and is characterized by stillbirth, diffuse generalized oedema within body cavities and tissues, under-developed lungs, and generalized hypoplasia of lymph tissues [1,2,3]. The enlarged fetus frequently causes dystocia and may require Cesarean section. Various forms of the disease have been reported in humans (OMIM:236750; OMIM:613124), mice models [4,5], and Online Mendelian Inheritance in Animals (OMIA) [6] lists entries for ‘pulmonary hypoplasia with anasarca’ or ‘hydrops fetalis’ in cattle (OMIA:001562-9913, OMIA:000493-9913), sheep (OMIA:000493-9940), rabbits (OMIA:000493-9986), dogs (OMIA:000493-9615), and pigs (OMIA:000493-9823).

In humans, hydrops fetalis is not considered a standalone disorder, but rather a feature of many disorders, with non-immune hydrops fetalis (NIHF; OMIM:236750) being the most frequent form of hydrops fetalis reported [7]. Causes of NIHF are often multifaceted and can include fetal and chromosomal abnormalities, metabolic disorders, congenital infections, lysosomal storage diseases, and rare genetic disorders [7,8]. Given the vast range of genetic etiologies for NIHF, the approach of categorizing the affected organs in addition to NIHF has meant that numerous genes of interest have been identified [9].

The genetic etiology of hydrops fetalis in rabbits and dogs is yet to be determined; however, a CRISPR-Cas9 experiment utilizing a 5-base pair (bp) deletion in the *FBN1* gene has resulted in piglets with hydrops fetalis phenotypes [10]. In mice, a genetic model involving a gene knockout of the *Adm* gene resulted in mice with similarly severe hydrops fetalis and cardiovascular defects [4]. In ruminants, PHA has been reported in Dexter (VBO:0000193), Shorthorn (VBO:00003750), Maine-Anjou (VBO:0000286), Cika (VBO:0005270), Holstein-Friesian (VBO:0000239), belted Galloway (VBO:0000142), and Ayrshire (VBO:0000120) cattle [1,2,11,12,13,14,15] and in several sheep breeds, including Cheviot (VBO:0001369), Merino (VBO:0001508), Poll Dorset (VBO:0001557)-cross, Awassi (VBO:0001308), and crossbred sheep [3,16,17,18,19]. The genetic cause has been established in Maine-Anjou and Dexter cattle: a point mutation and an 84 bp deletion, respectively [2]. However, the causal gene was not disclosed in these breeds despite the availability of a commercial test. In Cika cattle, a causative missense variant for PHA has been identified in the disintegrin and metalloproteinase with the thrombospondin type 1 motif 3 (*ADAMTS3*) gene located on chromosome 20 [12]. Häfliger et al. [12,13] also identified a trisomy of chromosome 20 in a Holstein fetus affected with PHA, and the resulting copy number variation of *ADAMTS3* was proposed to be disease causing.

Here, we report the clinical signs and pathology of four affected fetuses and the identification of a likely causal variant for PHA in Persian (VBO:0016885)/Persian-cross sheep.

## 2. Materials and Methods

### 2.1. Animals

Samples from three different Persian (VBO:0016855)/Persian-cross sheep flocks from Australia were available for this study.

Flock 1: One intact affected fetus was available for a complete necropsy (PPHA68). Additional tissue samples from four affected fetuses (PPHA69, PPHA70, PPHA93, and PPHA94) were collected by veterinarians and were available for histopathology only. Blood and tissue samples for DNA analysis were collected by veterinarians and/or the owner. EDTA blood samples were submitted from ten clinically normal dams or sires of affected lambs (obligate carriers) (PPHA73, PPHA74, PPHA79, PHA91, PPHA92, PPHA97, PPHA98, PPHA110, PPHA111, and PPHA112) and 20 other unaffected sheep that were not reported to be parents of an affected lamb (clinically unaffected). Tissue samples were available from ten affected fetuses (PPHA68, PPHA69, PPHA70, PPHA93, and PPHA94 collected for histopathology, as well as lung, liver, kidney and/or spleen from five additional fetuses PPHA72, PPHA100, PPHA-X, PPHA-Y, and PPHA-Z). Tissue samples were either formalin fixed, frozen fresh, or frozen in RNAlater (ThermoFisher Scientific, Waltham, MA, USA). Blood cards of 103 clinically unaffected animals were collected by the owner as per diagnostic DNA testing protocols (NSW Department of Primary Industries 2017). Pedigree information was provided by the owner from personal records and was visualized using R package kinship2 version 1.8.5 [20].

Flock 2: Cases of affected fetuses showing clinical signs similar to affected fetuses in flock 1 were historically observed. The owner of flock 1 had purchased several sheep from this flock. EDTA blood samples from one obligate carrier (PPHA21) and 65 clinically unaffected sheep were provided by the owner.

Flock 3: A formalin fixed paraffin embedded tissue sample of a single affected fetus (PPHA71) showing clinical signs similar to affected fetuses in flock 1 was provided by the owner.

### 2.2. DNA and RNA Extraction

Genomic DNA was extracted from 98 EDTA blood samples and 13 tissue samples using a QIAGEN DNeasy Blood & Tissue Kit (QIAGEN, Valencia, CA, USA) according to the manufacturer’s protocol. Formalin-fixed and paraffin-embedded tissues were treated using a tissue wash protocol [21] before extraction. Genomic DNA was isolated from blood cards collected from 103 animals using a standard blood card digest [22]. Total RNA was extracted from fresh spleen tissue stored in RNAlater (ThermoFisher Scientific, Waltham, MA, USA) from two affected animals (PPHA93 and PPHA94) using a QIAGEN RNeasy Mini Kit (QIAGEN, Valencia, CA, USA) following the manufacturer’s protocol for animal tissues.

### 2.3. Pathology

Detailed necropsy was conducted on one affected fetus (PPHA68). Tissue samples (skin, mesenteric lymph node, lung, thymus, liver, spleen, bone marrow, heart, kidney, adrenal gland, pancreas, salivary gland, small intestine, large intestine, skeletal muscle, visceral nerves, and brain) were collected and fixed in formalin. Additional formalin-fixed tissues of affected fetuses PPHA69 (lung), PPHA70 (brain), and PPHA93 and PPHA94 (lung, skin, mesenteric lymph node, liver, kidney, heart, spleen, gall bladder, and small and large intestine) were collected for histopathology by veterinarians. Formalin-fixed tissues were embedded in paraffin wax, sectioned at 5 microns, and then stained with haematoxylin and eosin.

### 2.4. SNP Genotyping

Genomic DNA samples from five affected fetuses (PPHA68, PPHA69, PPHA72, PPHA93, and PPHA94), five obligate carriers (PPHA21, PPHA73, PPHA91, PPHA92, and PPHA110), one suspected carrier (PPHA19, reported as possible sire of one affected lamb), and three clinically unaffected Persian/Persian-cross sheep (PPHA83, PPHA12, and PPHA13) as well as 22 control samples from different breeds (six Icelandic and 16 Merino sheep) were submitted to the Australian Genome Research Facility (AGRF) for genotyping with the Illumina^®^ OvineSNP50 Genotyping BeadChip (Neogen, Lansing, MI, USA). Runs of homozygosity (ROH) were computed using PLINK v1.07 [23] as previously described [24]. The ROH were visualized using R software v4.0.0 and Excel v16.0.

### 2.5. Whole Genome Sequencing

Concentration and purity of DNA from two affected fetuses (PPHA93 and PPHA94) and the obligate carrier dam (PPHA92) of PPHA94 were determined as previously reported [25]. DNA library preparation and sequencing were conducted by the Kinghorn Centre for Clinical Genomics (Sydney, NSW, Australia). An Illumina^®^ TruSeq DNA Nano Library Prep kit (Illumina, San Diego, CA, USA) was used for both affected animals, and a Roche KAPA PCR-Free Library Prep kit (Roche, Basel, Switzerland) was used for the obligate carrier animal. Samples were sequenced as 150 bp paired-end reads at an expected 30× coverage on an Illumina HiSeqTM X Ten sequencing platform (Illumina, San Diego, CA, USA). Adaptor sequences were removed, and FastQC (version 0.11.3) (https://www.bioinformatics.babraham.ac.uk/projects/fastqc/ (accessed on 25 September 2024)) analysis identified that the sequence data were of good quality (yield ranged from 58.37 GB to 76.23 GB, 82.45% to 96.6% > PHRED30, 40.5% to 42% GC content and no adaptor contamination) and did not require quality trimming.

### 2.6. Read Mapping, Variant Calling and Annotation

Analysis was conducted as previously reported [25]: Sequence reads were mapped to the Oar_v3.1 genome assembly (GCA_000298735.1) using Burrows–Wheeler Aligner (BWA-mem) version 0.7.15 [26]. PCR duplicates were marked with samblaster version 0.1.22 [27]. Lane-level BAMs were merged using Picard version 1.119 (http://broadinstitute.github.io/picard (accessed on 25 September 2024)). Sorting and indexing were performed with SAMtools version 1.6. Local realignment around insertion and deletion sites as well as base quality score recalibration using known variants downloaded from Ensembl’s dbSNP for Ovis aries version 87 [28] were executed with a Genome Analysis Toolkit version 3.7.0 (GATK) [29,30].

GATK HaplotypeCaller in GVCF mode [31], GATK GenotypeGVCFs [29,30], and SnpEff version 4.3 [32] were used for single nucleotide polymorphisms (SNP) calling, genotyping, and annotation and prediction of functional effects based on Ensembl annotation release 86 for Oar_v3.1.

### 2.7. Candidate Gene Analysis

Candidate genes were identified using searches in PubMed [33], Online Mendelian Inheritance in Man (OMIM) [34], and Online Mendelian Inheritance in Animals (OMIA) [6]. The terms ‘genetic OR congenital’, ‘lymphoedema’, ‘pulmonary hypoplasia’, and ‘hydrops (fetalis OR foetalis) AND pulmonary hypoplasia’ were used to search the PubMed database [33]. The term ‘non- immune hydrops fetalis’ was used to search the OMIM database [34], and the exhaustive search terms ‘hydrops foetalis’, ‘lymph node hypoplasia’, and ‘pulmonary hypoplasia’ were used for the OMIA database [6]. Functional candidate genes were prioritized based on their biological gene function and disease associations from OMIM [34], Mouse Genome Informatics (MGI) [35], and PubMed [33] to identify genes causing similar phenotypes to PHA.

### 2.8. Variant Filtering

Variants annotated by SnpEff [32] within a 40 megabase (Mb) region containing the functional candidate gene *ADAMTS3* (GCA_000298735.1, Chr6:87,097,877–87,386,270) were filtered using a case-control approach in SnpSift version 4 [36]. Variants that were homozygous alternate for affected fetuses PPHA93 and PPHA94 and not homozygous alternate for obligate carrier PPHA92 were identified and filtered for ‘low’, ‘moderate’, or ‘high’ impact on protein function. Known dbSNP variants and duplicate variants were removed. Remaining variants in *ADAMTS3* were visualized using SAMtools tview.

### 2.9. Validation of ENSOART00000014359.1:c.2055+3delG

PrimerBLAST [37] was used to design primers to amplify the region flanking the ENSOART00000014359.1:c.2055+3delG (Oar_v3.1) variant. PCR amplification of a 360 bp product was performed in one affected fetus (PPHA68), seven obligate carrier animals (PPHA21, PPHA73, PPHA74, PPHA79, PPHA91, PPHA92, and PPHA110), four clinically unaffected Persian/Persian-cross sheep (PPHA12, PPHA13, PPHA19, and PPHA83), and one Merino control using a Gradient Palm-Cycler™ Thermal Cycler (CGI-96, Corbett Life Science, Sydney, NSW, Australia). Each 25 μL reaction contained 1× Platinum™ SuperFi™ PCR Master Mix (Invitrogen, ThermoFisher Scientific, Waltham, MA, USA), 0.5 μM of primers F1 5′-AAAATTTTCTCCAGTGACCAGTTTA-3′ and R2 5′-CTTCCATCTTATACAGCCAAGAAAA-3′, and approximately 50 ng of genomic DNA. The PCR cycle included initial denaturation at 98 °C for 30 s, 40 cycles of 98 °C for 10 s, 64 °C for 10 s and 72 °C for 30 s, and a final extension at 72 °C for 5 min. PCR products were visualized on a 2% agarose gel and submitted to Macrogen (Seoul, Korea) for Sanger sequencing. Sequencing data were aligned to the Oar_v3.1 reference genome.

A database of known ovine variants generated by the Agriculture Victoria Research team at the Centre for AgriBioscience (Melbourne, VIC, Australia), was searched for the ENSOART00000014359.1:c.2055+3delG variant. The database includes variants discovered in whole genome sequencing from 935 sheep from 31 countries and 69 breeds including 21 Persian sheep from Iran (for details see European Variation Archive project: PRJEB31241) [38].

### 2.10. In Silico Prediction of Consequences of ENSOART00000014359.1:c.2055+3delG

To assess the strength of the 5′ splice site junction, a splice site motif scoring system based on an maximum entropy scoring model (MaxENT), MaxEntScan::score5ss (http://hollywood.mit.edu/burgelab/maxent/Xmaxentscan_scoreseq.html (accessed on 25 September 2024) [39], was used. A 9-mer sequence for the donor 5′ splice site of intron 14/15 was used for the ovine *ADAMTS3* wildtype (ADAMTS3_WT: gtgGTGAGT), the mutant splice site (ADAMTS3_MT: gtgGTAGTT), and a cryptic splice site identified in cDNA analysis (ADAMTS3_CT: tgtGTGCGT). As a comparison, the human wildtype (ADAMTS3_HUM_WT: gtgGTAAGT), mutant (ADAMTS3_HUM_MT: gtgGTAGTT), and cryptic (ADAMTS3_HUM_CT:tgtGTGCGA) equivalents of the ovine variant *ADAMTS3* were also tested for splice site strength. For these analyses, only the maximum entropy model was selected as the scoring model.

The JSI splice site prediction tool varSEAK (v.2.0; https://varseak.bio/index.php (accessed on 25 September 2024)) for human sequences was used to analyze the predicted impact of the equivalent variant in the human *ADAMTS3* transcript (ENST00000286657.4) using ADAMTS3_HUM_MT: gtgGTAGTTCAAATTGCTTCCCCAAAG as the input sequence.

### 2.11. In Vivo Consequences of ENSOART00000014359.1:c.2055+3delG

To assess the impact of the ENSOART00000014359.1:c.2055+3delG (Oar_v3.1) variant in cDNA, PrimerBLAST [37] was used to design primers to amplify the cDNA from exons 13 to 17 that contained the variant. A reverse transcriptase PCR (RT-PCR) amplification was performed in two affected sheep (PPHA93 and PPHA94) using a Gradient Palm-Cycler™ Thermal Cycler (CGI-96, Corbett Life Science, Sydney, NSW, Australia) in a total volume of 25 μL containing 10 μL of 2.5× OneStep Ahead RT-PCR Master Mix (QIAGEN, Valencia, CA, USA), 1 μL of 25× OneStep Ahead RT-Mix (25×) (QIAGEN, Valencia, CA, USA), 0.5 μM of primers F4 5′-TGAACATCCCGACTCCAAGAA-3′ and R4 5′-TTGACTTGGCTTCCTCCCCTT-3′, approximately 100 ng of total RNA from PPHA93 and PPHA94, and additional RNase-free water to the total volume per reaction. An initial denaturation step was performed at 50 °C for 10 min and then at 95 °C for 5 min. This was followed by 40 cycles at 95 °C for 10 s, 55 °C for 10 s, and 72 °C for 10 s. A final extension step was performed at 72 °C for 10 s. The RT-PCR products were visualized on a 2% agarose gel before submission to Macrogen (Seoul, Korea) for Sanger sequencing. Sanger sequencing results contained overlapping chromatogram peaks, and so a nested PCR was conducted to improve specificity. A primer pair was designed for a nested PCR using PrimerBLAST [37]. Primers were located in exon 14 and exon 17, and diluted RT-PCR product from both affected animals was used as template. Amplification was conducted in a Gradient Palm-Cycler™ Thermal Cycler (CGI-96, Corbett Life Science, Sydney, NSW, Australia) in a final volume of 25 μL containing 10 μL of 2.5× OneStep Ahead RT-PCR Master Mix (QIAGEN, Valencia, CA, USA), 1 μL of 25× OneStep Ahead RT-Mix (25×) (QIAGEN, Valencia, CA, USA), 0.5 μM of primers R1 5′-CGCGCTGTTCCTACAAAGAC-3′ and R4 5′-TTGACTTGGCTTCCTCCCCTT-3′, a 1/1000 dilution of the RT-PCR product, and additional RNase-free water to the total volume per reaction. An initial denaturation step was performed at 50 °C for 10 min and then at 95 °C for 5 min. This was followed by 40 cycles of 95 °C for 10 s, 55 °C for 10 s, and 72 °C for 10 s. A final extension step was performed at 72 °C for 10 s. The nested PCR products were visualized on a 2% agarose gel before submission to Macrogen (Seoul, Korea) for Sanger sequencing.

After cDNA analysis, the strength of the 5′ cryptic splice site junction for intron 14/15 resulting from the c.2055+3delG was investigated using MaxEntScan::score5ss (http://hollywood.mit.edu/burgelab/maxent/Xmaxentscan_scoreseq.html (accessed on 25 September 2024)) [39]. A 9-mer sequence for the donor 5′ splice site of intron14/15 was used for the ovine *ADAMTS3* wildtype (ADAMTS3_WT: gtgGTGAGT) and cryptic splice site (ADAMTS3_CT: tgtGTGCGT) cDNA. As a comparison, the human wildtype (ADAMTS3_HUM_WT: gtgGTAAGT) and mutant (ADAMTS3_HUM_CT:tgtGTGCGA) *ADAMTS3* cDNA (ENST00000286657.10) equivalent of the ovine variant was also tested for splice site strength. For both analyses, only the maximum entropy model was selected for the scoring model.

### 2.12. TaqMan PCR Genotyping Assay

A TaqMan real-time PCR was designed using the Custom TaqMan^®^ Assay Design tool (ThermoFisher Scientific, Waltham, MA, USA) to genotype the ENSOART00000014359.1:c.2055+3delG variant. The assay was performed using the ViiA™ 7 system (Applied Biosystems™, Foster City, CA, USA) in a total volume of 12.5 μL. Each reaction contained 1 x TaqMan^®^ Genotyping Master Mix (Applied Biosystems, Foster City, CA, USA), 900 nmol/L of primers 5′-ACTAGAGGAAGCTTCGGTGGAA-3′ and 5′-CAAAGACCCGTACAGCATATGTGT-3′, 250 nmol/L of allele specific 5′-VIC-TGTGTGGTGAGTTCAG-NFQ-3′ (wildtype) and 5′-FAM-AGTGTGTGGTAGTTCAG-NFQ-3′ (mutant) probes, and approximately 20 ng of genomic DNA. A pre-read stage at 60 °C for 30 s and an initial denaturation at 95 °C for 10 min were followed by 45 cycles of denaturation at 95 °C for 15 s, annealing/extension at 60 °C for 60 s, and a final post-read stage at 60 °C for 30 s. Data were analyzed using the QuantStudio™ Real-Time PCR System version 1.3 (Applied Biosystems™, Foster City, CA, USA).

## 3. Results

### 3.1. Clinical Signs and Pedigree Analysis

**Figure 1:**
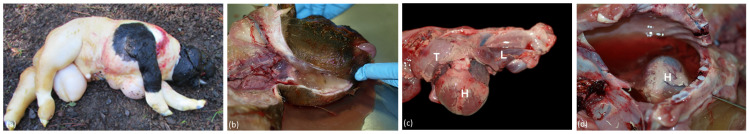
Gross morphology of PHA-affected fetuses reported in Persian/Persian-cross sheep. (**a**) An affected PHA fetus with severe malformation due to systemic oedema. (**b**) A submandibular midline incision showing subcutaneous fluid accumulation. (**c**) Thoracic viscera including left hypoplastic lung (L), heart (H), and thymus (T). (**d**) Right thorax with pleural fluid accumulation. Heart (H) is visible but extensive fluid obscures the hypoplastic lungs.

Several cases of abnormally large pregnant ewes with edematous fetuses leading to severe dystocia were reported in at least three flocks of Persian and Persian cross bred sheep from 2014 onwards. This is a relatively uncommon breed in Australia and the three flocks were known to be genetically linked. Clinical signs first manifested in pregnant ewes that showed bloating, lethargy, and recumbency. On vaginal examination of the ewes, it became apparent that the fetuses were profoundly large and edematous, requiring a caesarean section or euthanasia of the ewe. The affected fetuses showed systemic oedema with high volumes of pleural fluid and were stillborn (Figure 1).

Pedigree analysis of flock 1 supports the suspected recessive mode of inheritance for PHA in Persian sheep (Figure 5).

### 3.2. Gross Pathology

Full necropsy conducted on a female fetus (PPHA68) showed marked anasarca with subcutaneous oedema over the head, neck, thorax, and limbs (Figure 1). The fetus had a crown to rump length of 50 cm and a bodyweight of 9.7 kg. Moderate oedema was also observed in the caudal portion of the fetus, with patchy subcutaneous congestion and hemorrhage. No discernible lymph nodes were visible in the peripheral, mesenteric, or other abdominal regions. A small structure measuring 3 mm was observed in the parotid salivary tissue, although it could not be confirmed as a lymph node. The liver, kidneys, and spleen appeared normal. The abomasum contained a moderate amount of yellow mucoid fluid and there was mild reddening of the small intestinal serosa. The meconium of the large intestine was semi-fluid. Large quantities of yellow fluid were present in the thorax, and the lungs were bilaterally hypoplastic, with each lobe measuring approximately 25 mm in length, 20 mm in width, and 12 mm in depth. The heart appeared to be globose but otherwise normal. Excess translucent pericardial fluid was observed, as well as marked subserosal oedema of the parietal pleura. The thymus appeared normal, and the brain appeared mildly misshapen with an elongated appearance. The spine appeared fractured and separated in the upper lumbar region, with minor hemorrhage in adjacent soft tissue. In addition, mandibular prognathia was observed, with the lower incisor region extending beyond the dental pad.

For PPHA93 and PPHA94, samples for histopathology were collected by a veterinarian. At sample collection, large quantities of pleural fluid were observed in both PPHA93 and PPHA94. Necropsy of lung tissue from PPHA93 revealed small lung lobes measuring 55 mm in total length with the caudal lobe measuring 30 mm in length, 17 mm in width, and 17 mm in depth. Necropsy of PPHA94 showed small lungs similar to PPHA93, as well as a swollen liver with uniform orange discoloration. The gall bladder had collapsed with only trace bile present. Gross pathology was not available for PPHA69 and PPHA70, but preliminary diagnosis of all affected cases included pulmonary hypoplasia with anasarca with lymphoid hypoplasia and dysplasia, as well as cholangiopathy in some cases (PPHA68 and PPHA94).

### 3.3. Histopathology

**Figure 2:**
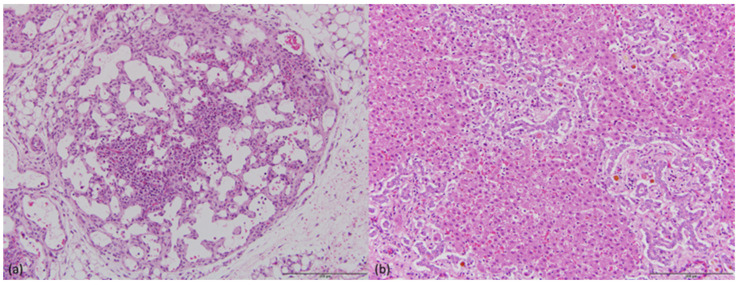
Histopathology of PHA-affected fetus PPHA68 tissues. (**a**) Dysplastic mesenteric lymph node lacking normal architectural arrangement. The lymphocytes are scant and are intermingled with granulocytes. (**b**) Liver with proliferative disorganized bile ductules and intraductular or periductular bile stasis. Scale bar = 200 μM.

Microscopic findings were similar in skin, lymph nodes, and lung of all fetuses sampled. In skin samples the deep dermis and subcutis were markedly expanded due to diffuse oedema. Expanded subcutaneous tissue contained sparse fine collagen fibers and plump pleomorphic spindle-shaped cells. In PPHA68, subcutaneous lymph vessels were prominent, dilated, and of irregular tortuous shape. The mesenteric lymph nodes were small and lacked normal architectural arrangement, consisting of clustered abnormal lymph vessels within a loose collagenous stroma. The stroma was scantly populated with lymphocytes and contained areas of extramedullary hemopoiesis (Figure 2a). In sections of lung the bronchi appeared smaller than normal, with lobes showing crowding of the bronchi, bronchioles, and large blood vessels. Bronchioles contained collapsed lumina with mucosal epithelium thrown into folds or papillary projections. Within alveoli there was patchy accumulation of foamy macrophages. A pleural, sub-mesothelial, moderately thick layer of fibrous tissue containing many lymph vessels and blood vessels was observed.

In the liver of PPHA68 and PPHA94, there was diffuse cholangiole proliferation in portal areas and moderate portal proliferation of plump spindle cells in a scant collagenous stroma resembling primitive mesenchyme (Figure 2b). In PPHA68, there was additionally marked stasis of the bile within canaliculi, particularly within portal connective tissue, and within some bile ductules. There was moderate diffuse cloudy swelling of hepatocytes in PPHA94. In the liver of PPHA93, there were no significant findings.

In the kidney of PPHA93 and PPHA94, cortical clusters of tubules of immature appearance alternated with areas of normal tubules. Glomeruli appeared to be concentrated in the outer cortical zone, with dilatation of the renal pelvis and moderate oedema of hilar connective tissue. There were no significant findings in the kidney of PPHA68.

There were no significant findings in the remaining tissues sampled from PPHA68, PPHA93, and PPHA94. As only the brain was available for PPHA70 and did not show any significant findings, no further observations could be made regarding other organ structures. The dams of PPHA69 and PPHA70 were also available for necropsy and a range of organs were examined histologically. The uterus was sampled from the dam of PPHA69 only and showed marked uterine and placental oedema. Both dams showed hyperplastic abomasitis typical of nematode parasitism, but significant changes were not detected in other organs.

### 3.4. Identification of Candidate Genes

Five protein coding genes were identified as functional candidate genes based on similar phenotypes to PHA observed in humans and mice (Table S1) [40,41,42.^43^,^44^,^45^,^46^]. These genes included: *ADAMTS3,* fms related receptor tyrosine kinase 4 (*FLT4*), forkhead box C2 (*FOXC2*), piezo type mechanosensitive ion channel component 1 (*PIEZO1*), and SRY-box transcription factor 18 (*SOX18*). The *ADAMTS3* gene has been implicated in lymphangiogenesis, and the proteins from the ADAMTS family are thought to have numerous functions within the extracellular matrix [40,47]. Variants in *ADAMTS3* cause Hennekam lymphangiectasia-lymphedema syndrome 3 in children [41] and upper airway syndrome due to airway oedema in dogs [48]. The FLT4 and FOXC2 genes are associated with lymphangiogenesis during early development [49,50]. Similarly, the PIEZO1 protein is involved in the mechanotransduction of several cell types, including the vasculature system. The SOX18 gene is involved in maintaining the endothelial barrier of cells, as well as vascular and lymphatic development and maintenance [46,51]. The *ADAMTS3* gene was considered a strong candidate gene based on its function in an *Adamts3*-/- knockout mouse model [48] and the ROH results in this study. The selection of *ADAMTS3* as a strong candidate gene was later supported by its genetic implication in PHA-affected cattle [12].

### 3.5. SNP Genotyping

Samples with call rates under 98% were removed from this study, which resulted in the removal of five animals, three of which were PHA-affected fetuses (PPHA68, PPHA69, and PPHA72) and two control Merino sheep. Call rates for the remaining 31 samples were on average 99.5%.

**Figure 3:**
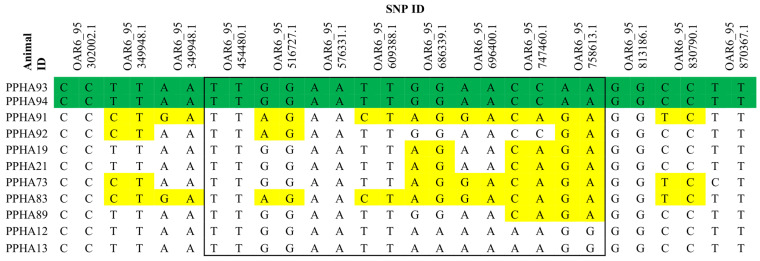
Schematic summary of SNP genotyping results for affected (PPHA93 and PPHA94), obligate carriers (PPHA91, PPHA92, PPHA21, PPHA73, and PPHA110 (shown with alternate ID PPHA89), suspected carrier (PPHA19), and clinically unaffected sheep (PPHA83, PPHA12, and PPHA13) Persian/Persian-cross animals on the Ovis_aries_1.0 genome assembly (GCA_000005525.1). The region containing the *ADAMTS3* gene is highlighted by a black box. A region of homozygosity is highlighted in green amongst affected animals, and heterozygous SNPs are highlighted in yellow.

The PHA-affected fetuses (PPHA93 and PPHA94) showed a shared region of homozygosity for approximately 40 Mb on chromosome 6, which contained the *ADAMTS3* gene. This region of homozygosity for *ADAMTS3* was not conserved in the obligate carrier or clinically unaffected Persian/Persian-cross animals (Figure 3). For the remaining four candidate genes, no shared regions of homozygosity were identified for the two PHA-affected fetuses.

### 3.6. Whole Genome Sequencing

Whole genome sequencing data for affected fetuses (PPHA93 and PPHA94) and one obligate carrier (PPHA92) identified 1958 raw variants in the SnpEff annotated VCF file in the *ADMATS3* positional candidate gene. After removal of known dbSNP variants and duplicates, only one variant was identified within the functional candidate gene *ADAMTS3* that was homozygous alternate in the affected fetuses and heterozygous in the obligate carrier PPHA92. This variant was a single nucleotide deletion predicted to be located within the exon14/intron14 splice site ENSOARG00000013204:g.87124344delC; ENSOART00000014359.1:c.2055+3delG (reverse strand) and was homozygous alternate in both affected fetuses (Table S2). Visual inspection of this variant using SAMtools tview [52] in PPHA93, PPHA94, and PPHA92 confirmed these genotypes.

### 3.7. Validation of c.2055+3delG

**Figure 4:**
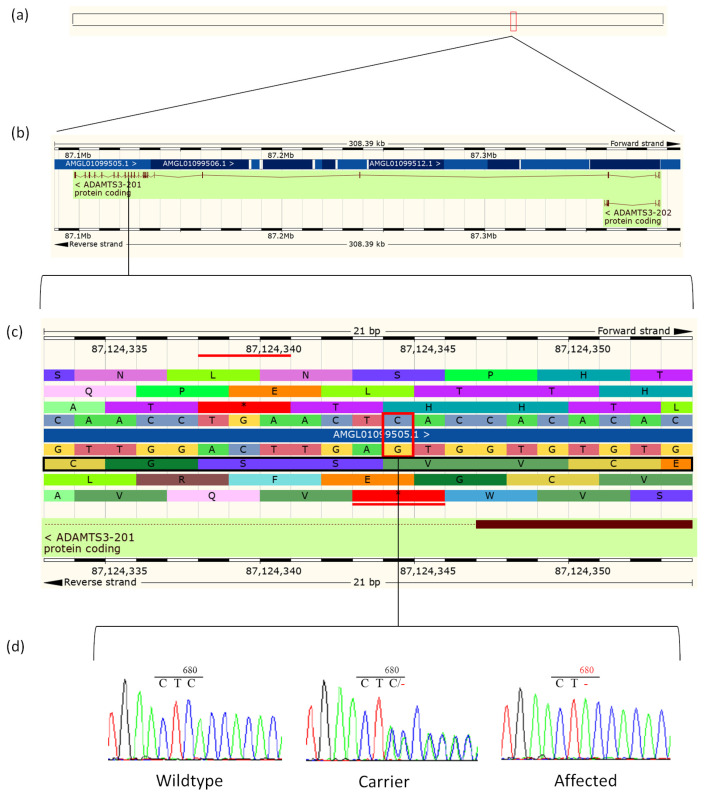
Ovine *ADAMTS3* and the ENSOARG00000013204:g.87124344delC variant. (**a**) Location of the ovine *ADAMTS3* gene (Chr6:87,097,877–87,386,270) on the Oar_v3.1 assembly. (**b**) Expanded view of the *ADAMTS3* gene showing all 22 exons. (**c**) Sequence level view of the region containing the g.87124344delC variant including protein translation frames (obtained from Ensembl, accessed 20 September 2020). The nucleotide that is deleted in affected animals is identified by a red box and the protein reading frame is marked with a black border. (**d**) Sequencing chromatograms for the wildtype (PPHA19), obligate carrier (PPHA92), and affected sheep (PPHA68).

Sanger sequencing of 13 animals, which included one affected fetus (PPHA68), seven obligate carrier animals (PPHA21, PPHA73, PPHA74, PPHA79, PPHA91, PPHA92, and PPHA110), four clinically unaffected Persian/Persian-cross sheep (PPHA12, PPHA13, PPHA19, and PPHA83) and one Merino control sheep, showed segregation of the variant with the PHA phenotype considering a recessive mode of inheritance. Affected fetus PPHA68 was homozygous alternate (-/-) for the deletion (Figure 4), all obligate carriers were heterozygous (C/-), and the four clinically unaffected Persian animals were heterozygous (PPHA83) or homozygous wildtype (C/C; PPHA12, PPHA13 and PPHA19) for the deletion. The Merino control was homozygous wildtype.

**Figure 5:**
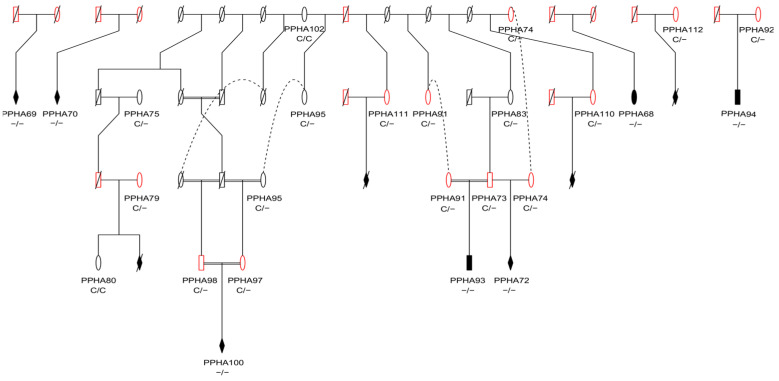
Pedigree of flock 1 with available genotypes showcasing inbreeding within the flock and segregation of the disease-causing variant. Males are designated by a square, and females are designated by a circle. Wildtype animals are designated by black outlines, obligate carriers are highlighted in red, and affected animals are designated by black-filled symbols. Symbols with a diagonal strike-through have not been genotyped or sequenced. Connecting dotted lines represent the same animal in other pedigree branches. Genotypes for the ENSOART00000014359.1:c.2055+3delG variant are shown as C/C, C/- or -/-.

Results from the TaqMan genotyping assay showed segregation of the variant with disease in the 209 Persian/Persian-cross sheep across flocks 1 and 2. All ten affected animals tested were homozygous alternate, all eleven reported obligate carriers were heterozygous, and the remaining animals were either homozygous wildtype (n = 116) or heterozygous (n = 72) (Table S3). The DNA quality for the affected animal from flock 3 was insufficient for the genotyping assay. The two Merino controls were homozygous wildtype. Based on pedigree information provided for flock 1, the c.2055+3delG variant segregates with disease (Figure 5). The estimated allele frequency for flocks 1 and 2 was 25%.

The c.2055+3delG variant was not listed as a known variant in the Ensembl Genome Browser and was not present in 935 whole genome sequenced sheep from 31 countries and 69 breeds [38].

### 3.8. Functional Consequences of ENSOART00000014359.1:c.2055+3delG

SnpEff identified ENSOART00000014359.1:c.2055+3delG as a splice region and intron variant at the boundary of exon 14 and intron 14/15 of the *ADAMTS3* gene. In silico analysis with MaxENT and varSEAK supports that the variant ENSOART00000014359.1:c.2055+3delG is predicted to disrupt the splice site. The MaxENT score for the wildtype sheep and human sequence was high, with a score of 8.95 and 10.36 respectively, while the mutated splice site in both the mutant ovine and the equivalent mutant human sequences resulted in the same weak scores for both sheep and human: MaxENT = 1.00. Using the varSEAK software, which is only available for human sequences, a human variant equivalent to the ovine variant was predicted to impart a loss of function of the authentic splice site and induce exon skipping, with a +71.01% reference score (wildtype) and a −64.02% variant score (mutant). A MaxENT score was also calculated using varSEAK of 10.36 for the wildtype human sequence and 1.00 for the human equivalent of the ovine variant.

**Figure 6:**
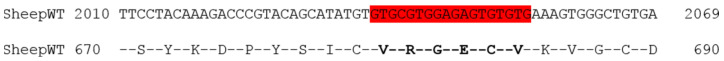
Schematic diagram of part of the ovine ADAMST3 cDNA and protein showing the location of the removed 18 nucleotides (highlighted in red) due to the variant ENSOART00000014359.1:c.2055+3delG and the predicted loss of six amino acids ENSOARP00000014152.1:p.(Val680_Val685del) in bold nucleotides. Figure adapted from Ensembl (accessed 12 September 2020).

Sanger sequencing of PCR products that were generated using RT-PCR amplicons from cDNA from two affected animals indicated that the ENSOART00000014359.1:c.2055+3delG variant results in the activation of a cryptic splice site within exon 14 for both PPHA93 and PPHA94. The resulting cDNA lacked the last 18 nucleotides of exon 14 (Table S4), and the protein is therefore predicted to contain 6 fewer amino acids (p.(Val680_Val685del)) (Figure 6, Figures S1 and S2). The loss of these 6 amino acids is located within the thrombospondin type 1 repeats (TSP1) conserved domain of the ADAMTS3 protein [28] (Figure S2). We propose that this deletion of 6 amino acids via the activation of a cryptic splice site is likely to be disease causing.

In silico analysis of this cryptic splice site (TGT|gtgcgt) using the MaxEnt splice site motif scoring system [39] did predict a lower MaxENT value for the cryptic splice site (MaxENT = 0.78) when compared to the mutant (MaxENT = 1) and wildtype (MaxENT = 8.95) sequences.

Affected fetus PPHA94 also appeared to present with an additional splice variant, as an alternative exon followed the shortened exon 14 sequence after analysis of the cDNA PCR products (Table S4). Analysis with the Basic Local Alignment Search Tool (BLAST) and Multiple Sequence Alignment Viewer (MSA) identified that the 139 bp sequence in the cDNA of PPHA94 was a possible alternative exon. From this sequence, 83 bp were identical to transcribed sequences in humans, mice, and dogs. The full 139 bp sequence was identified in flying foxes, mole rats, and some birds. The 139 bp sequence was not identified in annotated ovine transcripts (Figure S1) [53]. A BLAST search did not identify this sequence to be present in the ovine reference genome, possibly due to gaps in the genome. As this alternative exon appears to represent a normal splice variant in other species and it was only detected in one of the two affected fetuses, we are uncertain if it is associated with the disease.

## 4. Discussion

Our study identified a novel, likely causal variant, ENSOARG00000013204:g.87124344delC, in the *ADAMTS3* gene in PHA-affected fetuses by utilizing a multi-faceted approach. This approach harnessed homozygosity analysis of SNP genotyping data, whole genome sequencing and Sanger sequencing of both genomic and cDNA, and the development of a genotyping assay to aid in breeding management.

The PHA cases reported in Persian/Persian-cross sheep of this study are phenotypically similar to previously reported cases in sheep and cattle [1,2,3,11,12,13,14,15,16,17,18,19]. Similar clinical signs across these species include stillbirth, diffuse oedema, marked anasarca, lymph node hypoplasia, and dystocia. Hydrops fetalis in mice has so far only been observed in laboratory models, with the knockout of the adrenomedullin gene (*Adm*) being shown to produce hydrops fetalis phenotypes and cardiovascular abnormalities [4]. Hydrops fetalis in humans, however, is more difficult to clinically and genetically define due to its syndromic nature and association with a multitude of disorders [7]. However, acute oedema within body cavities [7] and cardiovascular abnormalities [9] are common clinical signs.

The inclusion of etiologic categories for NIHF by Bellini et al. [7] such as cardiovascular, chromosomal, extra thoracic tumors, gastrointestinal, hematologic, idiopathic, inborn errors of metabolism, infections, lymphatic dysplasia, miscellaneous, syndromic, thoracic, twin-to-twin transfusion-placental, and urinary tract malformations highlights the difficulty in attributing a correct diagnosis and targeting viable candidate genes for hydrops fetalis. Lymphoid hypoplasia, and at times complete absence of lymphoid tissue, is an important phenotypic hallmark that must be considered when selecting candidate functional genes. Five functional candidate genes *ADAMTS3*, *FOXC2*, *FLT4*, *PIEZO1*, and *SOX18* were selected in this study due to their involvement with lymphatic development. Of these candidate genes, it has been identified that biallelic loss of function variants in the *PIEZO1* gene can cause an autosomal recessive disorder, congenital lymphatic dysplasia with non-immune hydrops fetalis [54,55]. Similarly, both recessive and dominant variants in the *SOX18* gene in humans and mice showcase defective lymphatic tissue development followed by diffuse lymphatic oedema [51,56]. However, in PHA cases reported in cattle, a majority of causative variants have been identified in *ADAMTS3* ([12], Jonathan Beever, University of Tennessee, pers. comm.). The family of ADAMTS proteins play a role in cell–matrix interactions and the extracellular matrix, as well as some members being involved in embryogenesis and angiogenesis [40,57]. The ADAMTS3 protein is thought to play a role in fibrillary procollagen maturation; however, the function of this protein is still to be fully understood [57]. A knockout *Adamts3*-/- mouse model was generated to elucidate the function of *ADAMTS3* in vivo [40]. The *Adamts3*-/- embryos were not viable past day 15 of gestation; however, a reduced liver size, diffuse lymphoedema, and complete lack of lymphatic tissue were observed in these mice when compared to *Adamts3*+/+ embryos [40]. This obvious phenotype in *Adamts3*-/- mouse embryos added strong support for the selection of *ADAMTS3* as a candidate gene for PHA in sheep, given the highly similar phenotypes observed. In humans, variants in *ADAMTS3* cause the more benign Hennekam lymphangiectasia–lymphedema syndrome 3, characterized by polyhydramnios and congenital lymphedema of lower extremities [41]. In dogs, an even milder phenotype restricted to lymphedema of the upper airway has been associated with an *ADAMTS3* variant [48].

A majority of PHA cases in cattle have followed a recessive mode of inheritance, and this has also been predicted in sheep [2,12,19]. In the present study, the pedigree information for flock 1 is supportive of a recessive mode of inheritance (Figure 5). Pedigree analysis, combined with genotype analysis for flock 1, supports that the g.87124344delC variant segregates with disease.

The primary challenge when investigating rare inherited diseases is the availability of samples and suitable sample sizes for meaningful analyses. It is however possible to identify and prioritize positional candidate genes by first utilizing approaches such as SNP genotyping and identifying runs of homozygosity within small sample sizes [58]. When using this approach, it is particularly important to have clear phenotype descriptions and flock history. Despite using only two of five affected animals for SNP genotyping analysis due to DNA quality issues, a region of homozygosity was able to be identified when coupled with obligate carrier and clinically unaffected sheep, and this approach allowed for prioritization of one of the functional candidate genes identified for further analysis. Whole genome sequencing rather than direct sequencing of the *ADAMTS3* gene was considered more cost effective as the *ADAMTS3* gene is relatively long with a gene length of 1,711,607 bp (ENSOARG00000013204) and a transcript length of 4120 bp (ENSOART00000014359.1) and 1138 bp (ENSOART00000014360.1) for the two transcripts currently annotated. Further, this data type would have enabled us to investigate other functional candidate genes should our selected candidate gene have not yielded a plausible candidate variant.

The c.2055+3delG variant identified in this study is predicted to disrupt the 5′ donor splice site in intron 14/15 of *ADAMTS3* and was shown to result in the activation of a cryptic splice site resulting in a loss of six amino acids (p.(Val680_Val685del)) (Figure 6). The assessment of the strength of this mutant splice site using maximum entropy scores [39] for both the ovine and human sequences showed that the low score of the mutant splice site in both the ovine and human sequences support that the c.2055+3delG variant disrupts the splice site.

Larger numerical values for the MaxENT score are typically associated with more efficient splicing and, therefore, a stronger splice site for each exon [59]. It has been reported that the ideal MaxENT score for a 5′ donor splice site in human sequences is 11.81 [59]. Both the wildtype MaxEnt scores for the ovine and human intron 14/15 donor splice site were strong at values of 8.95 and 10.36, respectively. The MaxENT scores for the mutant splice sites had a low value of 1.0. However, the proposed activated cryptic splice site identified via sequencing of cDNA had an even lower value of 0.78. Other consequences in addition to the activation of the cryptic splice site, such as exon skipping, can therefore not be excluded. The prediction of the equivalent mutant splice site in humans using varSEAK suggested a class five splicing effect that resulted in a loss of function. The in silico predictions, the cDNA analysis, the segregation of the variant with disease, and the evidence of similar phenotypes in cattle and mice resulting from variants in the *ADAMTS3* gene do support that this variant is disease-causing, however not all consequences of the disruption of the splice site may have been discovered.

This study was limited by the availability of tissues and RNA from affected, obligate carrier, and clinically unaffected animals to further analyze the functional effect of the splice variant. If high quality RNA from affected animals from different tissues and time points becomes available, RNA sequencing analysis or quantitative RT-PCR could be used to further analyze splice variants and expression in this gene and their association with disease. Variants in splice site regions have been shown in humans [60] and animals to cause disease, with 126 splicing variants currently listed in OMIA as deleterious variants for Mendelian traits [6]. Variants in splice site regions are especially important when the exon/intron boundary sequence is altered from the standard GT dinucleotide at the 5′ end of the exon [60,61]. Despite the difficulty in identifying splice site variants and their effect on gene isoform structure [62], numerous acceptor splice site variants have been identified as causal in human and animal inherited diseases.

The loss of six amino acids (p.(Val680_Val685del)) is located within the TSP1 conserved domain of the ADAMTS3 protein (Figure S2). The TSP1 domain is part of the extracellular matrix of a wide variety of cells and functions as an inhibitor to endothelial cell growth and angiogenesis. It has been shown in cattle that the TSP1 domain binds latent TGF-β, which is produced by cells in a latent form [63]. This latent form of TGF-β is actively involved in angiogenesis and other biological processes [63]. The deletion of six amino acids within the TSP1 domain could impact on the ability for TSP1 to bind latent TGF-β; however, further in silico and in vitro protein structure and function analysis is required to fully elucidate the impact of the c.2055+3delG variant on protein function.

Interestingly, there was evidence of an alternative exon 15 in the cDNA of PPHA94. As this alternative exon was not detected in the cDNA of PPHA93, it is unlikely to be disease-related, but may present a tissue specific splice variant as only RNA from spleen tissue was available for this study.

The use of a SNP genotyping and whole genome sequencing approach has allowed for the identification of g.87124344delC as a likely disease-causing variant. This has enabled the development and successful use of a genotyping assay (described in Section 2.12) to identify heterozygous animals in flock 1. Since the implementation of genotyping, no further affected fetuses have been observed in the flock as the owner is now able to avoid carrier by carrier matings. Wider use of DNA testing for this variant will enable for improved breeding management and animal welfare in Persian/Persian-cross sheep in Australia.

## 5. Conclusions

This study is the first report of PHA in Persian/Persian-cross sheep in Australia. We have described the pathology in affected fetuses and identified a novel variant, ENSOARG00000013204:g.87124344delC, in the positional and functional candidate gene *ADAMTS3* as a likely causal variant for PHA. A diagnostic DNA test was developed and allows for effective breeding management of this recessive condition. Our clinical and pathological findings have provided further insight into the function of the ADAMTS3 protein.

## Data Availability

The dataset generated and/or analyzed during the current study are available at the European Nucleotide Archive (http://www.ebi.ac.uk/ena/ (accessed on 25 September 2024)) and was deposited under the study accession number PRJEB39179, with sample accession numbers SAMEA7034588, SAMEA7034589, and SAMEA7034590.

## Supplementary Materials

The following supporting information can be downloaded at: https://www.mdpi.com/article/10.3390/ani14192811/s1, Figure S1: Overview of the *ADAMTS3* cDNA sequence using the NCBI Multiple Sequence Alignment Viewer (version 1.16.1) across multiple species. The 139 bp sequence was not identified in annotated ovine transcripts. Figure S2: Ensembl overview of the ADAMTS3 protein with conserved domains between the (**a**) wildtype protein and the (**b**) mutant protein. The mutant protein shows a shorter overall amino acid length when compared to the wildtype protein length, with the loss of six amino acids in the thrombospondin type 1 repeats. Table S1: Top five protein coding functional candidate genes identified based on literature review of similar phenotypes to PHA in humans and mice. Table S2: List of 44 private whole genome sequencing variants identified in two affected fetuses (PPHA93 and PPHA94) and one obligate carrier (PPHA92) after filtering based on segregation, predicted protein impact, removal of known SNPs, and duplicates. Table S3: TaqMan PCR assay genotyping results for 212 animals. Table S4: Overview of exon length in the *ADAMTS3* gene in humans (Homo sapiens), mice (Mus musculus), sheep (Ovis aries), and PHA-affected fetuses PPHA93 and PPHA94. Data for affected sheep is based on sequencing of cDNA generated using primers in exon 13 and 17. Affected sheep have a shortened exon 14 and normal length exon 15 and 16.
