# Supplementary material for: A Splice Site Variant in ADAMTS3 Is the Likely Causal Variant for Pulmonary Hypoplasia with Anasarca in Persian/Persian-Cross Sheep
Source: Animals (Basel). 2024 Sep 29;14(19):2811. doi: 10.3390/ani14192811 (PMC11475510; doi:10.3390/ani14192811)
Supplement: Supplementary file 1 [file animals-14-02811-s001.zip › animals-3199457-supplementary/Supplementary_Figures S1 - S2.pdf]

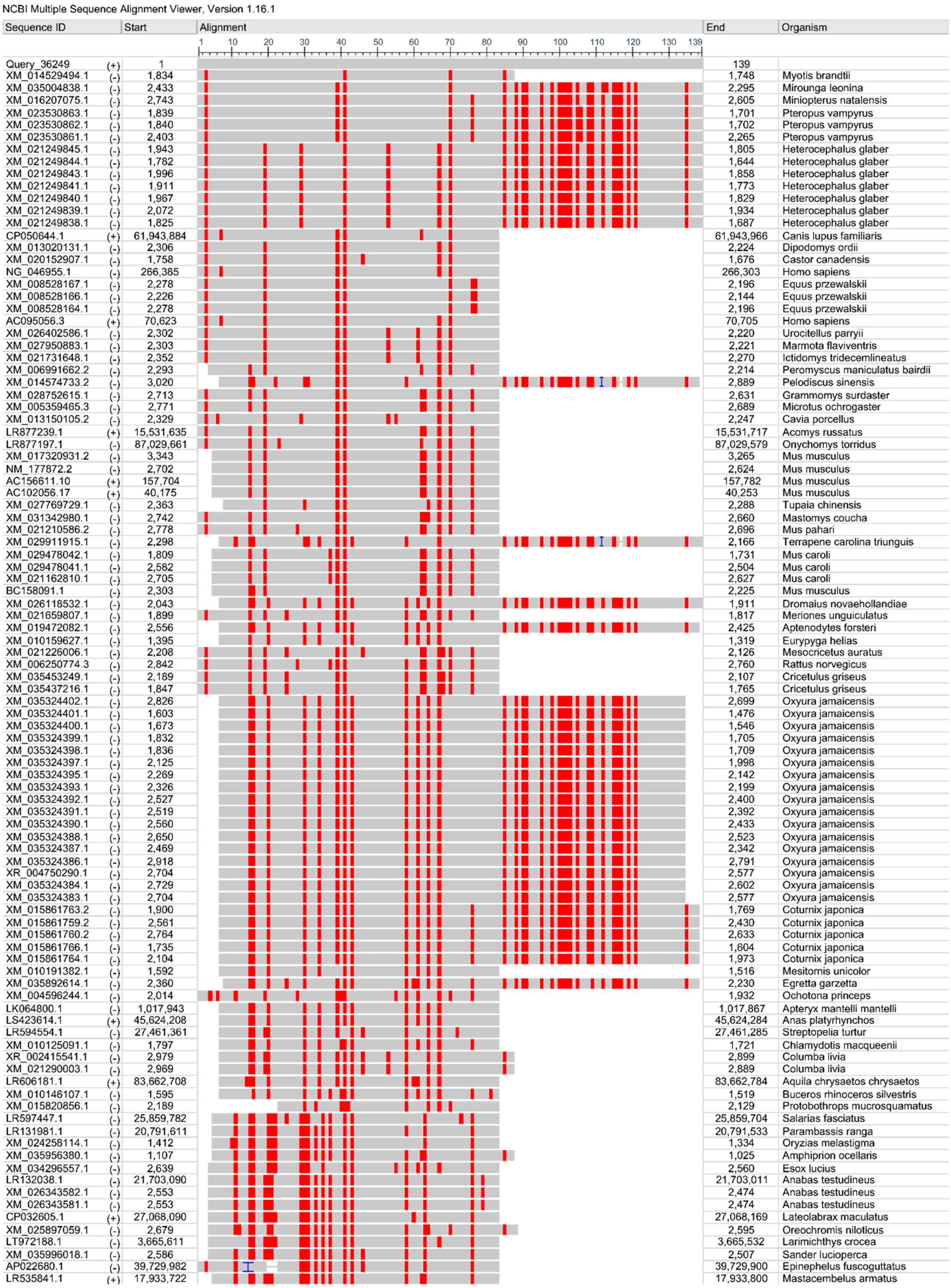

Figure S1. Overview of the ADAMTS3 cDNA sequence using the NCBI Multiple Sequence Alignment Viewer (version 1.16.1) across multiple species. The 139 bp sequence was not identified in annotated ovine transcripts.

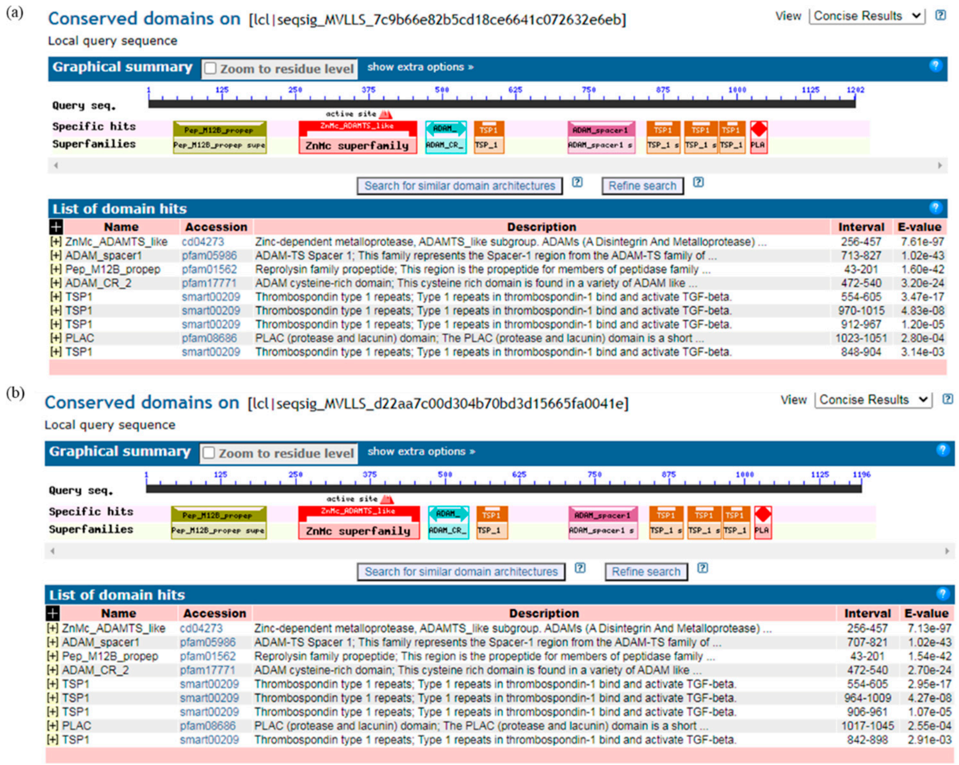

Figure S2. Ensembl overview of the ADAMTS3 protein with conserved domains between the (a) Wildtype protein and the (b) Mutant protein. The mutant protein shows a shorter overall amino acid length when compared to the wildtype protein length, with the loss of six amino acids in the thrombospondin type 1 repeats.
